# Correction to: HDAC5, Negatively regulated by miR‐ 148a‐ 3p, promotes colon cancer cell migration

**DOI:** 10.1111/cas.16125

**Published:** 2024-02-27

[OuYang C, Shu G, Liu J, et al. HDAC5, negatively regulated by miR‐148a‐3p, promotes colon cancer cell migration. *Cancer Sci*. 2022;113:2560–2574. https://doi.org/10.1111/cas.15399.]

In the initially published version of this article, there were issues with the images of si‐DAC5/invasion in Figure 2D and HDAC5/migration in Figure 2G. The corrected Figure 2 is shown below. These corrections will not have any impact on the main findings of the article.

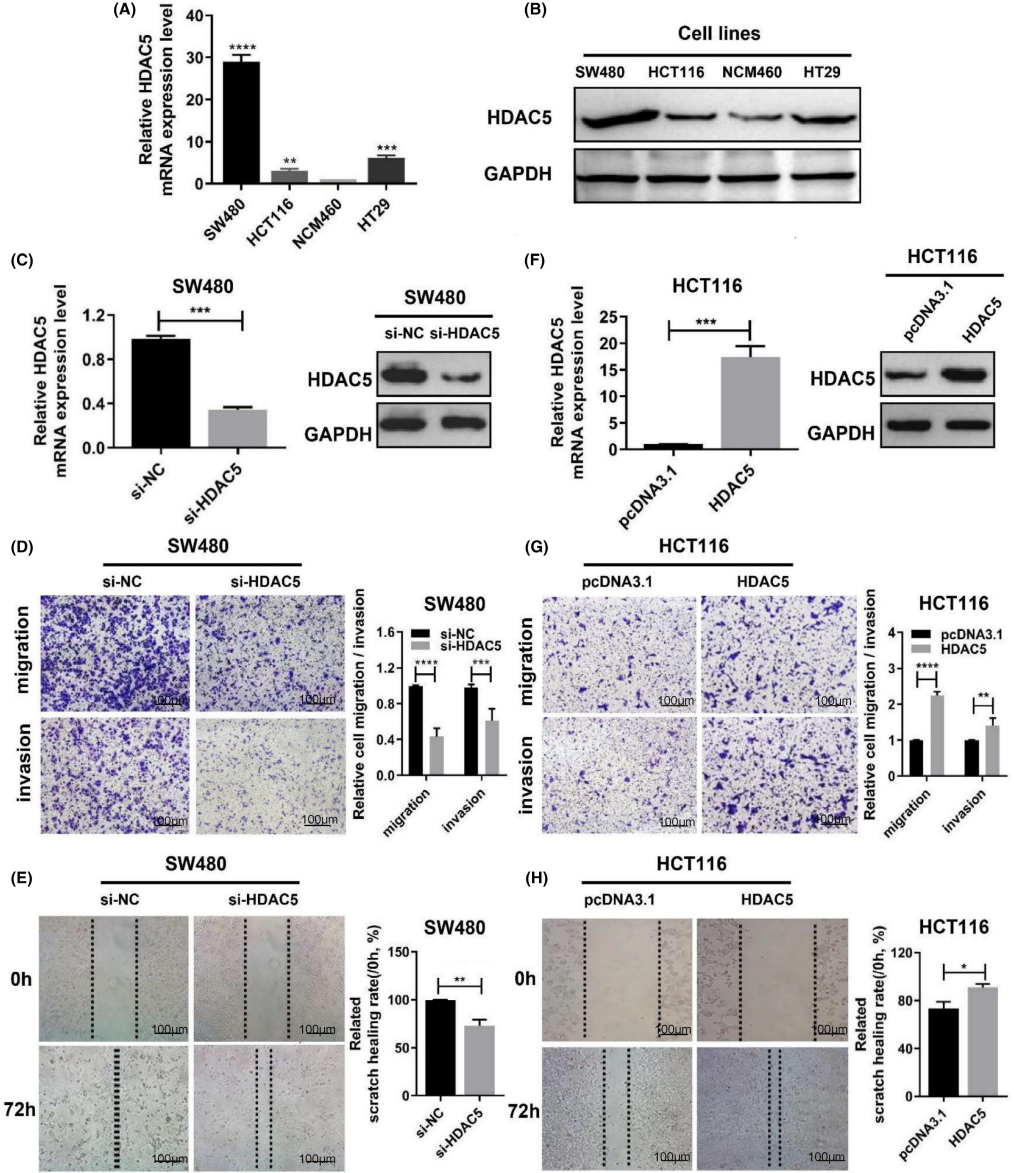

We apologize for this error.

